# High Prevalence of Overweight/Obesity in Urban Sri Lanka: Findings from the Colombo Urban Study

**DOI:** 10.1155/2019/2046428

**Published:** 2019-11-22

**Authors:** Noel Somasundaram, Ishara Ranathunga, Kavinga Gunawardana, Muneer Ahamed, Dileepa Ediriweera, C. N. Antonypillai, Nishan Kalupahana

**Affiliations:** ^1^Diabetes and Endocrine Unit, National Hospital of Sri Lanka, Colombo, Sri Lanka; ^2^Diabetes and Endocrine Unit, Teaching Hospital, Kandy, Sri Lanka; ^3^Faculty of Medicine, University of Kelaniya, Sri Lanka; ^4^Department of Physiology, Faculty of Medicine, University of Peradeniya, Sri Lanka

## Abstract

**Background:**

South Asian countries face a double burden of malnutrition characterized by high prevalence of underweight, overweight, and obesity. Understanding the distribution of this public health problem is important to tailor targeted interventions for communities. The objective of the current study was to find out the prevalence of obesity in urban Sri Lanka and to identify sociodemographic factors associated with it.

**Methods:**

Adult males and females residing in an urban government division of the Colombo District in Sri Lanka were included in this study (Colombo Urban Study). Stratified simple random sampling was used to select a sample of 463 from the total population. Sociodemographic data using an interviewer-administered questionnaire, anthropometric measurements, and serum samples were obtained for investigations.

**Results:**

When the global BMI cutoffs were applied, the community prevalences of underweight, normal weight, overweight, and obesity were 7.7%, 39.6%, 37.0%, and 15.8%, respectively. When the Asian BMI cutoffs were applied, the respective prevalences were 7.7%, 26.8%, 34.3%, and 31.2%. The community prevalence for abdominal obesity was 58.1% when using Asian cutoffs. Females had a higher prevalence of both obesity and abdominal obesity. There was an ethnic difference in obesity rates with Moors having the highest rates (65.5%) followed by Sinhalese (52.3%) and Tamils (40.2%). The highest obesity prevalence was observed in the most educated group. Multiple regression analysis showed that high BMI was associated with female gender and family history of hypertension. Serum LDL negatively associated with BMI while the strength of this relationship was impacted by serum HBA1c levels. Finally, serum triglyceride level showed positive association with BMI, and the effect was more marked in Moors compared to Sinhalese.

**Conclusion:**

Two-thirds of adults in the studied urban population were overweight or obese. This highlights the urgent need for interventions to curb this epidemic. The gender, ethnic differences in obesity, its associations with educational status, and the interactions with metabolic comorbidities indicate that these interventions may need to be targeted towards different groups in the population.

## 1. Background

Global obesity prevalence has increased from 3·(2%) in 1975 to 10·(8%) in 2014 in men and from 6·(4%) to 14·(9%) in women [1]. While obesity was considered to be a major public health issue in the developed world [2], recent data from different countries show that there is a progressive increase in obesity rates within each country [1]. When considering developing countries, although infectious diseases and undernutrition are still major health concerns, the prevalences of obesity and related issues are on the rise [3]. Sri Lanka, which is a South Asian country with a population of 20 million, has been experiencing rapid and sometimes unplanned urbanization and infrastructure developments over the recent past with an estimated 30% of the population now living in urban and suburban areas [4]. There has also been economic growth from low income to lower-middle income.

A national level study done in 2005/2006 by Katulanda et al. reported the percentages of Sri Lankan adults having overweight, obesity, and abdominal obesity as 25.2%, 9.2%, and 26.2%, respectively [5]. However, no recent data is available about the obesity rates in Sri Lanka. Moreover, data on the obesity prevalence among the urban population in Sri Lanka is unavailable. A recent survey in India showed a higher obesity prevalence among the urban population compared to rural population [6]. It is important to find out whether similar patterns are seen in Sri Lanka as well, since it will be important to tailor targeted interventions for obesity prevention. Furthermore, in Sri Lanka, there is still a significant burden of underweight especially among the rural population [7]. Indeed, South Asia is facing a double burden of malnutrition [8]. Therefore, when devising public health interventions to tackle the double burden of obesity and underweight, it is critical to have an understanding of the distribution of these problems in the country.

The recent escalation of obesity rate is attributed to a change in lifestyle, characterized by consumption of energy-dense unhealthy food and engaging in sedentary behaviors. Moreover, low calcium intake and low vitamin D status are also associated with obesity [8]. While the aforementioned factors and their link with obesity have been extensively studied in Western populations, there is paucity of such data from South Asian countries. A recent study from Sri Lanka found an association between physical activity and weight status while no such association was found with food habits [9]. Since the dietary and other lifestyle patterns are different in Sri Lankans compared to other countries [10], it is important to identify the association of these factors with obesity. While there are evidence-based strategies to prevent obesity and other noncommunicable diseases [11], identification of the lifestyle and sociodemographic factors associated with obesity will help to tailor interventions to curb the obesity rates in Sri Lanka.

The current study was done as there is lack of recent data available for the prevalence of obesity in urban Sri Lanka. Furthermore, available studies have not identified lifestyle and sociodemographic factors associated with obesity. The main objective of our study was to find out the prevalence of obesity in urban Sri Lanka and to identify lifestyle and sociodemographic factors associated with it.

## 2. Methods

The study population for this study was adult males and females who were 18 years and above, whose permanent residences were in the Eastern Kuppiyawatta local government (Grama Niladhari (GN)) division of the Colombo District. This local government area was selected for the community cohort as it is the closest to the National Hospital of Sri Lanka, which was the main research center.

### 2.1. Sample Size

Sample size was calculated using the Lwanga and Lameshow 1991 formula of *n* = *z*^2^ *p* (100 − *p*)*D*/*d*^2^. Sample size of 600 was calculated for an expected prevalence of obesity of 50%, with a design effect of 1.2, a precision of 95%, and an anticipated 25% nonresponse rate using the EPI 6 sample calculation software.

### 2.2. Sampling Technique

Stratified simple random sampling was used to select a sample of 463 from the total population of 6473 in the GN area in three strata in the age categories of 18-40 years, 40-60 years, and above 60 years. In order to ensure the precision of the estimates in the subsample analysis (according to the age groups), the sample was divided among the 3 age categories on a weighted basis that took into account the proportion in the population and the expected prevalence of obesity. Using a random number generator, study subjects were randomly selected into the three strata as follows. In the 18-40 years strata, 210 were selected (35% of total sample); in the 40-60 years strata, 240 were selected (40% of the sample); and in the above 60 years strata, 150 were selected (25% of the sample). The resulting disproportionate sample allocation was accounted for by the use of weighted analysis. The weights were the inversion of the sampling fractions in the analysis.

### 2.3. Data Collection

The participants were recruited at their homes by a team of researchers to provide an invitation letter and information documents. On the day of the screening, informed written consent was taken and data was collected using interviewer-administered questionnaire by trained interviewers. The data including sociodemographic data, use of alcohol, smoking, food frequency and physical activity, and detailed medical history on previous diagnoses and treatment were obtained. Anthropometric measurements were measured (weight, height, waist circumference, total body fat estimation, and visceral fat percentage using a bioimpedance analyser—OMRON HBF 516). The following data were measured in nine to twelve hours of fasting stage: plasma glucose (GOD-PAP5 method, Olympus AU 480/680/400 analyser), cholesterol (CHOD-PAP method, Olympus AU 480/680/400 analyser), triglyceride (GPO-PAP method, Olympus AU 480/680/400 analyser), glycosylated haemoglobin (HPLC method, Bio-Rad Variant II Turbo analyser), serum corrected calcium (Arzenso III method, Olympus AU 480/680/400 analyser), and 25-OH vitamin D level (direct chemiluminescence method, Advia centaur analyser). In nondiabetic patients, 75 g anhydrous glucose was given and blood was collected for glucose level two hours later.

### 2.4. Statistical Analysis

Data analysis was performed in the R programming language version 3.2.2 [12]. Community-based weight index (BMI category) prevalences with 95% confidence intervals for the urban study population and for different strata including age and gender were calculated considering the stratified sampling methodology using the “Survey” package in the R programming language [13]. Descriptive data analysis was done and tabulated to present study population characteristics. Exploratory data analysis was done to identify the variables associated with body mass index, and the variables studied were age, gender, ethnicity, education level, smoking habits, alcohol consuming habits, family history of diabetes, hypertension and hyperlipidaemia, fasting blood sugar, total cholesterol, low-density lipoprotein cholesterol (LDL), high-density lipoprotein cholesterol (HDL), triglycerides (TG), haemoglobin A1c (HbA1c), thyroid stimulating hormone (TSH), serum calcium, and vitamin D.

Initially, each study variable was screened with simple linear regression, and the variables significant at *p* = 0.2 level were subsequently used for multiple variable analysis with multiple linear regression. Significant variables at multiple variable analysis were selected for the final model, and interactions between variables were studied. The study variable ethnicity had 4 categories (i.e., Sinhalese, Tamils, Moors, and Other) where the “Other” ethnicity had only 4 individuals and this group was not considered in reporting prevalence rates and analyzing interaction at the final model. *p* value of 0.05 was considered as significant.

### 2.5. Ethical Issues

Ethical approval was obtained from the ethical review committee of the Faculty of Medicine, University of Colombo.

## 3. Results

**Figure 1:**
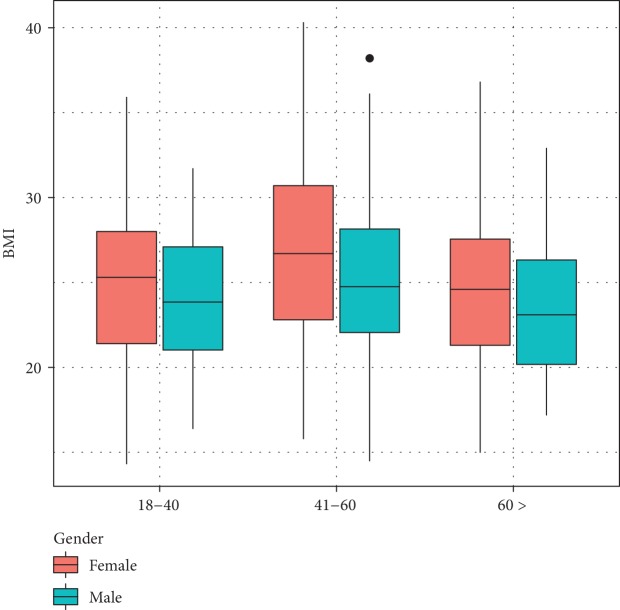
Distribution of BMI according to gender.

**Figure 2:**
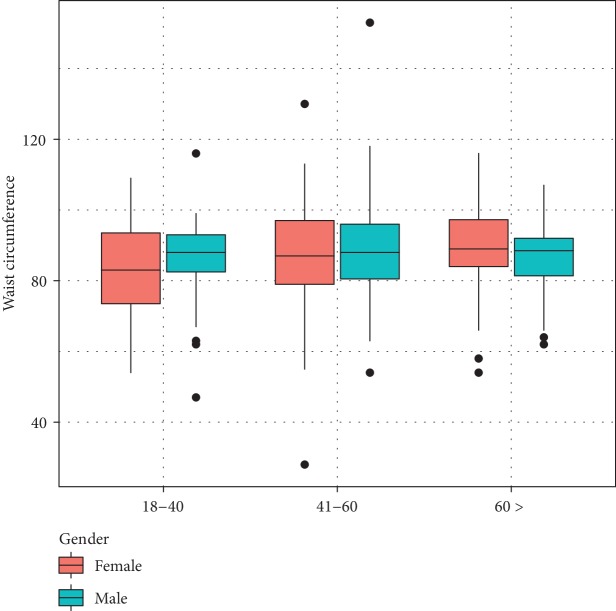
Distribution of waist circumference according to gender.

A total of 600 individuals were invited, and 463 subjects gave informed consent and completed the screening. Most of the respondents were females (69%). There were 124 in the 18-40 age group, and 70% of these were females. There were 209 respondents in the 41-60 age group, and 73% of these strata were females. In the over 60-year age strata, there were 130, and 63% were females. The response rate in each of the above strata was 59%, 87%, and 87% with an overall response rate of 77%.  Table 1 summarizes the basic characteristics of the study group. Figures 1 and 2 show the distribution of BMI and waist circumference of the age groups of study strata by gender.

When the global BMI cuffs were applied, the community prevalences of underweight (BMI < 18.5 kg/m^2^), normal weight (BMI 18.5-24.9 kg/m^2^), overweight (BMI 25-29.9 kg/m^2^), and obesity (BMI ≥ 30 kg/m^2^) were 7.7%, 39.6%, 37.0%, and 15.8%, respectively (Table 2). When the Asian BMI cutoffs [14] were applied, the prevalences of underweight, normal weight (BMI 18.5-22.9 kg/m^2^), overweight (BMI 23-27.49 kg/m^2^), and obesity (BMI ≥ 27.5 kg/m^2^) were 7.7%, 26.8%, 34.3%, and 31.2% (Table 2). Since South Asians are at a higher risk of metabolic comorbidities of obesity at a given BMI compared to Caucasians, the current recommendation for BMI cutoffs for this region are <18.5 kg/m^2^, 18.5-22.9 kg/m^2^, 23-27.49 kg/m^2^, ≥27.5 kg/m^2^ for underweight, normal weight, overweight and obesity respectively [15]. Using these cutoffs, the prevalences of underweight, normal weight, overweight, and obesity were 7.6%, 26.8%, 12.7%, and 52.8%, respectively (Table 2). Underweight among males was 8.1%, while it was 7.4% among females. Overweight and obesity rates in males were 14.5% and 44.6%, respectively, while 11.9% and 56.3% in females, respectively. When different age categories were considered, the highest obesity prevalence (58.3%) was found in the 41-60 years group, while the minimum prevalence (43.1%) was found among subjects older than 60 years (Table 2). The prevalences of obesity classes 1, 2, and 3 were 36.9%, 13.8%, and 1.9%, respectively (Table 3). Females had higher prevalence for all three obesity classes compared to males.

According to the International Diabetes Federation cutoff values on waist circumference for determining abdominal obesity (WC—male ≥ 90 cm and female ≥ 80 cm) [16], community prevalence for abdominal obesity was 58.1% (Table 4). Although there was increasing prevalence seen across higher age categories, among the overall community as well as among females, the age category with the minimum prevalence of abdominal obesity among males was >60 years. When the obesity rates among ethnic groups were considered, prevalences of overweight, obesity, and abdominal obesity were the lowest in Tamils and were the highest in Moor community (Table 5). Obesity rates were the highest in the most educated group. Interestingly, abdominal obesity was the highest in the least and most educated groups. Obesity seems to be higher in nonsmokers and nonconsumers of alcohol (Table 5). This could be due to a higher number of females being nonconsumers (42% of men were current or exsmokers compared with 2% in women) and obesity being higher among females.

### 3.1. Explorative Analysis

Next, we explored the factors associated with BMI in our sample. Initial individual variable analysis showed that gender, ethnicity, alcohol consumption, family history of diabetes, hypertension and hyperlipidaemia, level of fasting blood sugar, LDL, TG, HbA1c, calcium, and vitamin D were significantly associated with BMI.

Subsequent multiple variable analysis showed that gender, ethnicity, family history of hypertension, LDL, TG, and HbA1c were significantly associated with BMI and significant interaction existing between LDL and HbA1c as well as ethnicity and TG (Table 6). Males had a lower BMI compared to females, and individuals with a family history of hypertension had a higher BMI compared to individuals without family history of hypertension. TG, BMI, and ethnicity showed a complex interaction where TG levels increased along with the increasing BMI and the amount of increase depended on the ethnicity. Moors showed a comparatively higher increase in TG along with BMI compared to Sinhalese. There was no difference in TG increase among Tamils compared to Sinhalese and Moors. BMI, LDL, and HbA1c showed a complex interaction where LDL levels reduced along with the increasing BMI and the LDL reduction depended on the HbA1c levels. LDL reduction was high at lower HbA1c levels compared to higher HbA1c levels. Final multiple linear regression model showed overall significance (*F* = 9.06, df1 = 10, df2 = 442, *p* < 0.01) with multiple *r*-squared of 0.17 and adjusted *r*-squared of 0.15.

## 4. Discussion

The aim of the current study was to find out the prevalence of obesity and its associated sociodemographic factors in urban Sri Lanka. This study was carried out in a local government division of Colombo, which is the most urbanized district in the country. According to the WHO Asian obesity cutoffs, the community prevalences of overweight, obesity, and abdominal obesity were 34.3%, 31.2%, and 58.1%, respectively. Katulanda et al., from their national level study in 2005/2006, reported figures of 25.2%, 9.2%, and 26.2% for the same categories [5]. There could be two main reasons which may be responsible for the dramatic increase in obesity between the previous study and the current one. Firstly, the current study only considered an urban population of Colombo. Secondly, it is possible that obesity rates have risen in Sri Lanka similar to the other countries of the region, during the last decade. Indeed, similar high obesity rates have been reported in other countries of the region. For example, Nepal has an obesity prevalence of 32% [17].

When the South Asian obesity cutoffs were used, overweight and obesity were seen in 14.5% and 44.6% (total 59.1%) of males and 11.9% and 56.3% (total of 68.2%) of females, respectively. Abdominal obesity prevalence was 40% in males and 66.6% females. The International Day for the Evaluation of Abdominal Obesity (IDEA) study [18] also found similar high prevalence for abdominal obesity in South Asia (males 58% and females 75%) which was even worse than the situation in Northwest Europe. Also, the higher obesity and abdominal obesity rates seen in females in the current study are with similar trends in India, where obesity is more prevalent in females [19]. When considering different age categories, the highest prevalence for obesity of 58.3% was seen in 41-60 years group and the minimum prevalence of 43.1% among subjects older than 60 years (South Asian cutoffs applied). A similar pattern was seen in the previous study with regard to different age groups [5].

There was an ethnic difference in obesity rates in the current study. The prevalence of obesity was the highest among the Moor ethnicity (65.5%) followed by Sinhalese (52.3%) and Tamils (40.2%). A similar trend was seen in the prevalence of abdominal obesity (74.4%, 56.8%, and 44.8% in Moors, Sinhalese and Tamils, respectively). A study conducted in Kalutara district in Sri Lanka (a mixture of urban and rural communities) reported similar findings with the highest obesity rates among the Moor ethnicity [20].

We observed the higher prevalence of obesity in the most educated group (above A/L). However, abdominal obesity was higher in the least and most educated groups. In a recent systematic review on “educational attainment and obesity,” De Silva et al. reported that there is an inverse association between obesity rates and educational attainment in high-income countries, while there was a positive association between these variables in low-income countries [21]. The finding that the abdominal obesity rates are the highest among low and highest educational levels in our study could be due to the ongoing process of nutrition transition in Sri Lanka, where there is a higher availability of energy dense foods, with increasing sedentary behaviors specially among “white-collar” jobs.

Obesity seems to be higher in nonsmokers (*p* = 0.14) and nonconsumers of alcohol (*p* = 0.04). This could be due to a higher number of females being nonconsumers and obesity being higher among females (*p* < 0.01). But more importantly, 42% of males and 2.1% females were ex- or current smokers and 59.4% of males and 2.5% females were ex- or current alcohol consumers.

Elevated serum triglyceride level is a feature of metabolic syndrome [22], which is causally linked to abdominal obesity. Accordingly, we found a positive association between serum triglycerides and BMI (*p* < 0.01). Interestingly, there was an interaction between TG and ethnicity, with Moors showing a higher increase in TG with BMI compared to Sinhalese. It will be interesting to see whether a similar relationship will be seen in the reduction in TG with weight loss, which might be important in the management of these patients. Finally, we found a negative association between BMI and serum LDL level (*p* = 0.06). Interestingly, this relationship was the highest in individuals with the highest HBA1c level.

While we have identified the obesity prevalence and several variables associated with BMI, there are several limitations in the current study. We used multiple linear regression to identify the factors associated with BMI. We considered only the measured covariates and did not consider any nonlinear associations in the analysis; as a result, the model had a lower *r*-squared value (Table 6). This final model has lower predictive capacity as our analysis was not aimed at developing a predictive model for BMI. In the current study, we studied the obesity prevalence and factors associated with it in an urban sample from a local government area close to the main research center. Further, we could not study the suburban or rural populations.

## 5. Conclusions

Our study indicates that the majority of our study population is obese and/or centrally obese and that the prevalence is on the rise. Since this is an important risk factor for several of the noncommunicable diseases, it is timely if not late to actively look into this issue to intervene at all possible levels to prevent as well as to treat obesity. This study indicates that certain ethnic groups, females, and individuals with higher educational status are at a higher risk of obesity. We have previously reviewed potential public health interventions to prevent NCDs in South Asia [11]. In light of the current findings, these interventions may need to be targeted more towards the above high risk groups in the urban population.

## Figures and Tables

**Table 1:** Study group characteristics.

	Both sexes (*n* = 463)	Males (*n* = 143)	Females (*n* = 320)
Mean age (SD)	50.4 (14.8)	50.9 (15.8)	50.2 (14.3)
Ethnicity			
Sinhala	320 (69.1%)	108 (75.5%)	212 (66.2%)
Tamil	56 (12.1%)	12 (8.4%)	44 (13.8%)
Moor	83 (17.9%)	23 (16.1%)	60 (18.8%)
Other	4 (0.8%)	—	4 (1.2%)
Education			
Below grade 5	77 (16.7%)	10 (7.1%)	67 (20.6%)
Up to O/L	240 (51.9%)	77 (53.8%)	163 (51.1%)
Up to A/L	127 (27.5%)	47 (32.9%)	80 (25.1%)
Above A/L	18 (3.9%)	9 (6.2%)	9 (2.8%)
Tobacco smoking			
No	396 (85.6%)	83 (58.0%)	313 (97.9%)
Current smokers	40 (8.6%)	34 (23.8%)	6 (1.8%)
Exsmokers	27 (5.8%)	26 (18.2%)	1 (0.3%)
Alcohol consumers			
No	370 (79.9%)	58 (40.6%)	312 (97.5%)
Current consumers	71 (15.3%)	64 (44.7%)	7 (2.2%)
Exconsumers	22 (4.8%)	21 (14.7%)	1 (0.3%)
Diabetes			
No	155 (33.5%)	45 (31.4%)	110 (34.4%)
Prediabetes	150 (32.4%)	43 (30.1%)	107 (33.4%)
Diabetes	158 (34.1%)	55 (38.5%)	103 (32.2%)
Family history of diabetes	199 (43.0%)	64 (44.8%)	135 (42.2%)
Family history of hypertension	209 (45.1%)	67 (46.9%)	142 (44.4%)
Family history of dyslipidaemia	117 (25.3%)	31 (21.7%)	86 (26.9%)
Mean total cholesterol (SD)	210.3 (47.8)	205.1 (40.3)	212.7 (50.7)
Mean low-density lipoprotein (SD)	124.8 (43.1)	118.5 (35.3)	127.7 (45.9)
Mean high-density lipoprotein (SD)	59.2 (8.0)	59.0 (8.1)	59.3 (8.0)
Mean triglyceride (SD)	135.3 (64.7)	144.8 (66.7)	131.1 (63.4)
Mean HbA1c (SD)	6.4 (1.7)	6.5 (1.6)	6.4 (1.8)
Mean TSH (SD)	2.5 (7.1)	1.0 (4.1)	2.7 (8.2)
Mean calcium level (SD)	2.2 (0.1)	2.2 (0.01)	2.2 (0.1)
Mean vitamin D level (SD)	20.6 (7.0)	23.2 (8.2)	19.5 (6.1)
Mean waist circumference (SD)	87.0 (±13.0)	87.5 (±12.9)	86.8 (±13.0)
Mean BMI (SD)	25.2 (±4.8)	24.1 (±4.4)	25.7 (±4.8)

**Table 2:** Prevalence (with 95% confidence intervals) of weight status according to global, South Asian, and Asian BMI cutoffs.

	Underweight	Normal	Overweight	Obesity
According to global BMI cutoffs (*n* = 463)				
Both sexes (*n* = 463)				
All age groups	7.7 (4.9-10.4)	39.6 (34.8-44.4)	37.0 (32.2-41.8)	15.8 (12.3-19.3)
18-40	9.7 (4.4-15.1)	38.9 (30.1-47.8)	38.9 (30.1-47.8)	12.4 (6.4-18.4)
41-60	5.9 (2.8-9.0)	35.8 (29.5-42.1)	34.8 (28.5-41.1)	23.5 (17.9-29.1)
60 >	6.3 (2.6-9.9)	50.7 (43.1-58.3)	36.8 (29.5-44.1)	6.2 (2.6-9.9)
Males (*n* = 143)				
All age groups	8.1 (3.5-12.8)	47.2 (38.2-56.2)	36.7 (28.0-45.3)	8.0 (3.0-13.0)
18-40	5.6 (-1.8-12.9)	50.0 (33.9-66.1)	36.1 (20.7-51.6)	8.3 (0-17.2)
41-60	10.3 (2.8-17.9)	41.4 (29.2-53.6)	37.9 (25.9-49.9)	10.3 (2.8-17.9)
60 >	10.4 (2.4-18.5)	52.1 (38.9-65.2)	35.4 (22.8-48.0)	2.1 (0-5.8)
Females (*n* = 320)				
All age groups	7.4 (4.0-10.8)	36.2 (30.5-41.8)	37.1 (31.3-42.9)	19.3 (14.7-23.8)
18-40	11.7 (4.6-18.8)	33.8 (23.4-44.2)	40.3 (29.5-51.0)	14.3 (6.6-22.0)
41-60	4.1 (1.0-7.2)	33.6 (26.2-40.9)	33.6 (26.2-40.9)	28.8 (21.7-35.8)
60 >	4.2 (0.4-7.9)	50.0 (40.7-59.3)	37.5 (28.5-46.5)	8.3 (3.2-13.5)
According to Asian BMI cutoffs				
Both sexes (*n* = 463)				
All age groups	7.7 (4.9-10.4)	26.8 (22.4-31.2)	34.3 (29.6-39.0)	31.2 (26.7-35.8)
18-40	9.7 (4.4-15.1)	29.2 (20.9-37.5)	33.6 (25.1-42.2)	27.4 (19.3-35.5)
41-60	5.9 (2.8-9.0)	22.1 (16.6-27.5)	32.8 (26.4-39.0)	39.2 (32.8-45.7)
60 >	6.3 (2.6-9.9)	31.9 (24.9-34.0)	39.6 (32.2-47.0)	22.2 (15.9-28.5)
Males (*n* = 143)				
All age groups	8.1 (3.5-12.8)	32.7 (24.1-41.2)	35.1 (26.6-43.5)	24.1 (16.5-31.8)
18-40	5.6 (-1.8-12.9)	41.7 (25.8-57.5)	30.1 (15.7-45.4)	22.2 (8.9-35.6)
41-60	10.3 (2.8-17.9)	19.0 (9.3-28.7)	39.7 (27.6-51.8)	31.0 (19.6-42.5)
60 >	10.4 (2.4-18.5)	37.5 (24.8-50.2)	37.5 (24.8-50.2)	14.6 (5.3-23.9)
Females (*n* = 320)				
All age groups	7.4 (4.0-10.8)	24.2 (19.2-29.3)	33.9 (28.3-39.5)	34.4 (28.8-40.0)
18-40	11.7 (4.6-18.8)	23.4 (14.1-32.7)	35.01 (24.6-45.6)	29.9 (19.8-39.9)
41-60	4.1 (1.0-7.2)	23.3 (16.7-29.9)	30.1 (23.0-37.3)	42.5 (34.8-50.2)
60 >	4.2 (0.4-7.9)	29.2 (20.7-37.6)	40.6 (31.5-49.8)	26.0 (17.9-34.2)
According to South Asian BMI cutoffs				
Both sexes (*n* = 463)				
All age groups	7.6 (4.9-10)	26.8 (22.4-31.2)	12.7 (9.6-15.9)	52.8 (47.8-57.7)
18-40	9.7 (4.3-15.1)	29.2 (20.9-37.5)	9.7 (4.3-15.1)	51.3 (42.3-60.4)
41-60	5.8 (2.7-8.9)	22.1 (16.6-27.5)	13.7 (9.1-18.2)	58.3 (51.8-64.8)
60 >	6.2 (2.6-9.9)	31.9 (24.9-39.0)	18.7 (12.8-24.7)	43.1 (35.5-50.6)
Males (*n* = 143)				
All age groups	8.1 (3.5-12.7)	32.6 (24.1-41.2)	14.5 (8.6-20.4)	44.6 (35.7-53.6)
18-40	4.1 (0.0-12.9)	41.6 (25.8-57.5)	8.3 (0.0-17.2)	44.4 (28.5-60.4)
41-60	10.3 (2.8-17.8)	18.9 (9.2-28.6)	22.4 (12.1-32.7)	48.3 (35.9-60.6)
60 >	10.4 (2.3-18.5)	37.5 (24.8-50.2)	14.6 (5.3-23.9)	37.5 (24.8-50.2)
Females (*n* = 320)				
All age groups	7.4 (4.0-10.8)	24.4 (19.2-29.3)	11.9 (8.2-15.6)	56.3 (50.5-62.2)
18-40	11.6 (4.6-18.7)	23.4 (14.1-32.7)	10.4 (3.7-17.1)	54.5 (43.5-65.5)
41-60	4.1 (1.0-7.2)	23.3 (16.7-29.9)	12.3 (5.5-15.0)	62.3 (54.7-69.9)
60 >	4.1 (0.4-7.8)	29.2 (20.7-37.6)	20.8 (13.2-28.4)	45.8 (36.6-55.1)

**Table 3:** Prevalence (95% confidence interval) of the degree of generalized obesity.

	Obesity class 1 25 ≥ BMI < 30	Obesity class 2 30 ≥ BMI < 5	Obesity class 3 BMI ≥ 35
Both sexes (*n* = 463)			
All age groups	36.9 (32.2–41.8)	13.8 (10.4–17.2)	1.9 (0.7–3.2)
18-40	38.9 (30.1–47.8)	11.5 (5.7–17.3)	0.1 (0.0–0.2)
41-60	34.8 (28.5–41.1)	20.0 (14.8–25.4)	3.4 (1.1–5.8)
60 >	36.8 (29.5–44.1)	4.8 (1.5–8.1)	1.3 (0.0–3.1)
Males (*n* = 143)			
All age groups	36.6 (28.0–45.3)	6.1 (1.4–10.7)	1.8 (0.0–3.9)
18-40	36.1 (20.6–51.5)	8.3 (0.0–17.2)	0.0 (0.0–0.0)
41-60	37.9 (25.9–49.9)	5.1 (0.0–10.6)	5.1 (0.0–10.6)
60 >	35.4 (22.8–47.9)	2.1 (0.0–5.8)	0.0 (0.0–0.0)
Females (*n* = 320)			
All age groups	37.1 (31.3–42.3)	17.2 (12.9–21.6)	2.0 (0.1–0.3)
18-40	40.3 (29.4–51.0)	12.9 (5.5–20.3)	1.3 (0.0–3.7)
41-60	33.6 (26.2–40.9)	26.1 (18.2–32.8)	2.7 (0.1–5.2)
60 >	37.5 (28.5–46.5)	6.2 (1.7–10.7)	2.1 (0.0–4.7)

**Table 4:** Prevalence (95% confidence interval) of abdominal obesity.

	Normal	Abdominal obesity
Both sexes (*n* = 463)		
All age groups	41.9 (36.0–46.8)	58.1 (53.1–62.9)
18-40	46.8 (37.7–60.0)	53.2 (44.0–62.3)
41-60	38.4 (32.0–44.9)	61.6 (55.1–70.3)
60 >	37.1 (29.7–44.4)	62.9 (55.6–70.3)
Males (*n* = 143)		
All age groups	60.3 (41.4–69.0)	40.0 (31.0–48.6)
18-40	58.3 (42.5–74.2)	41.7 (25.8–57.5)
41-60	57.6 (45.5–70.0)	42.4 (30.2–54.5)
60 >	70.8 (58.9–82.8)	29.2 (17.2–41.1)
Females (*n* = 320)		
All age groups	33.6 (27.8–39.4)	66.4 (60.6–72.2)
18-40	41.3 (30.4–52.3)	58.7 (48.0–69.6)
41-60	30.6 (23.3–37.8)	69.4 (62.2–76.7)
60 >	20.0 (12.5–27.5)	80.0 (72.5–87.5)

**Table 5:** Prevalence (95% confidence intervals) of weight status according to ethnicity, education, smoking, and alcohol use (*n* = 463).

	Underweight	Overweight	Obesity	Abdominal obesity
Ethnicity				
Sinhala	9.3 (5.6–13.0)	10.9 (7.5–14.4)	52.3 (46.4–58.3)	56.8 (50.8–62.7)
Tamil	2.8 (0.0–8.2)	19.3 (9.3–29.3)	40.2 (26.3–54.2)	44.8 (30.8–58.8)
Moor	4.3 (0.0–9.2)	14.9 (6.3–23.4)	65.5 (54.3–76.7)	74.4 (63.8–85.0)
Education				
< grade 5	4.8 (0.2–9.3)	13.1 (5.2–21.0)	50.9 (39.3–62.4)	71.6 (60.7–82.6)
Up to O/L	8.7 (4.7–12.6)	12.5 (8.3–16.7)	54.5 (47.6–61.3)	55.3 (48.5–62.2)
Up to A/L	7.0 (1.8–12.2)	13.5 (7.3–19.7)	49.7 (40.2–59.1)	55.3 (45.8–64.8)
Above A/L	8.4 (0.0–23.8)	10.8 (0.0–26.6)	56.8 (31.9–81.7)	70.1 (47.7–92.5)
Smoking				
No	7.1 (4.2–10.0)	12.2 (8.8–15.6)	53.0 (4.8–58.3)	60.6 (55.3–65.9)
Current	13.3 (1.8–24.8)	12.6 (3.6–21.5)	47.2 (30.2–64.2)	40.4 (23.8–57.0)
Past	7.8 (0.0–16.4)	22.3 (7.3–37.4)	57.8 (38.8–76.8)	45.1 (24.4–65.8)
Alcohol				
No	7.6 (4.5–10.8)	11.9 (8.4–15.4)	54.1 (48.6–59.6)	61.9 (56.5–67.4)
Current	8.4 (1.2–15.6)	14.3 (6.7–21.9)	46.6 (33.9–59.4)	44.0 (31.4–56.7)
Past	5.4 (0.0–12.4)	23.5 (5.7–41.2)	46.2 (23.5–69.0)	31.1 (9.8–52.3)

**Table 6:** Multivariate regression for obesity with sociodemographic factors.

	Estimate	Std. error	*t* value	Pr (>∣*t*∣)
Intercept	18.83	2.42	7.79	<0.01
Male sex	-1.86	0.45	-4.14	<0.01
Ethnicity—Tamils	-1.99	1.61	-1.23	0.21
Ethnicity—Moors	-0.18	1.43	-0.13	0.89
Fh_ht	1.36	0.42	3.26	<0.01
LDL	0.03	0.02	1.47	0.14
TG	0.01	0.01	2.19	0.02
HBA1C	1.02	0.34	2.99	<0.01
LDL : HBA1C	-0.01	0.01	-2.48	0.01
Ethnicity—Tamils: TG	0.01	0.01	0.98	0.32
Ethnicity—Moors: TG	0.02	0.01	2.21	0.03

## Data Availability

The data analyzed in this paper can be made available to researchers. Requests for access to the data set used in this paper should be directed to the corresponding author.

## Abbreviations

BMI: Body mass index
LDL: Low-density lipoprotein cholesterol
HDL: High-density lipoprotein cholesterol
TG: Triglycerides
HBA1c: Haemoglobin A1c
TSH: Thyroid stimulating hormone
WHO: World Health Organization
WC: Waist circumference.
